# Marginal adaptation of different monolithic zirconia crowns with horizontal and vertical finish lines: A comparative in vitro study

**DOI:** 10.34172/joddd.2023.40589

**Published:** 2023-12-30

**Authors:** Mohammed Qasim Nasir, Alaa Jawad kadhim

**Affiliations:** Department of Restorative and Aesthetic Dentistry, College of Dentistry, University of Baghdad, Baghdad, Iraq

**Keywords:** Chamfer, Marginal gap, Monolithic, Vertical, Zirconia

## Abstract

**Background.:**

This study evaluated the influence of different tooth preparation techniques and zirconia materials on marginal adaptation.

**Methods.:**

Forty-eight healthy human maxillary first premolars were divided into two primary groups based on preparation design: group A (chamfer) and group B (vertical). Within each main group, there were three subgroups, comprising eight teeth each, distinguished by the type of zirconia material employed (Zircad LT, MT, and Prime by Ivoclar Vivadent). All the samples were prepared by the same operator using a dental surveyor. Intraoral scanning was performed on the prepared teeth. SironaInLab CAD 20.0 software was used to design crowns, which were subsequently generated using a 5-axis milling machine. The crowns were cemented to their respective teeth with self-adhesive resin cement. Marginal gap measurements were taken in micrometers (μm) before and after cementation at 16 sites per sample using a digital microscope at×230 magnification. The collected data were evaluated using statistical analysis using the independent t-test, paired t-test, and ANOVA at an 0.05 significance level.

**Results.:**

The vertical preparation group exhibited the smallest marginal gap, while the chamfer group displayed the largest. This disparity was statistically significant (*P*<0.05) for pre- and post-cementation measurements across all materials. There were no significant differences between the different monolithic zirconia crowns.

**Conclusion.:**

The vertical preparation design illustrated significantly better marginal adaptation than the chamfer preparation design. Comparisons between materials showed comparable marginal gaps. The mean values of the marginal gaps in all groups increased significantly after cementation.

## Introduction

 All-ceramic restorations have gained popularity in dentistry because of their enhanced biocompatibility and esthetics.^1^

 The marginal gap between the restoration and the prepared tooth causes bacterial aggregation, periodontal issues, recurrent caries, and restoration failure.^2^ Misfit, on the other hand, was found to reduce the fracture strength of ceramic restorations.^3^

 The tooth preparation design is an important component in the marginal accuracy and fracture strength of the restoration. As a result, the geometry and amount of tooth reduction should reduce stress and provide the best marginal fit to protect the health of the surrounding tissue and maximize the longevity of the restoration.^4^

 Since zirconia is opaque, it is frequently coated with porcelain in clinical conditions.^5^ Monolithic zirconia restorations have been introduced as an alternative to mitigate veneering porcelain chipping.^6^ The development of translucent zirconia materials, known as monolithic translucent zirconia, has overcome the poor optical characteristics of traditional zirconia.^5^ Traditional horizontal preparation with shoulder finish lines and chamfer has been the norm or standard for all-ceramic restorations. On the other hand, these preparations are invasive and require the removal of intact tooth structure, which is unfavorable for biological and aesthetic reasons.^7^ With the emergence of high-strength polycrystalline materials, vertical preparation has been proposed as an alternative that is less invasive than horizontal preparation.^8^

 Therefore, this research study examined the marginal adaptation of different monolithic zirconia materials in horizontal and vertical preparation techniques. The null hypothesis posited that the type of zirconia and preparation technique would not significantly affect marginal adaptation.

## Methods

 Forty-eight human maxillary first premolars chosen for this study were extracted from orthodontic patients aged 18‒22. Using G*Power 3.1.9.7 (a program developed by Franz-Faul at the University of Kiel in Germany), the sample size was determined with the following parameters: the power of the study = 95%, alpha error of probability = 0.05, a statistical test of analysis of variance, the effect size of F = 0.31, number of groups = 6, etc. With all these parameters met, the sample size was n = 42 plus a 10% error rate, making 48 samples sufficient for this study. To minimize variables, the teeth were evaluated for crown size using a digital caliper^9^ and evaluated using a digital microscope (Dino-Lite Capture 2.0, version 1.3.6., Taiwan) at × 40 magnification to exclude any teeth with caries, restorations, or cracks. The use of extracted human teeth was ethically approved by the Research Ethics Committee of the College of Dentistry, University of Baghdad (Project No.: 503522, Ref. No.: 503). To prevent fungal and bacterial infection, thymol solution is used at room temperature for one week,^10^ followed by immersion in distilled water to prevent the teeth from dehydration.^11^

 The individual teeth were placed in a custom-made square rubber mold measuring 2.0 cm in height, 1.5 cm in length, and 1.5 cm in width, filled with freshly mixed cold-cured acrylic resin. Next, a dental surveyor was used to ensure that each tooth was vertically aligned with the mold’s horizontal plane. To mimic the support of healthy alveolar bone, the teeth were embedded 2 mm apical to the cementoenamel junction (CEJ).^12^ The teeth were then categorized into two groups of 24, based on one of the preparation techniques (horizontal or vertical).

 Group A: Horizontally prepared teeth (chamfer finish line)

 Group B: Vertically prepared teeth

 Then, based on the type of material, each main group was subcategorized into three subgroups of eight teeth.

 Subgroup 1: Monolithic zirconia (IPS e.maxZirCAD LT) (IvoclarVivadent: Schaan, Liechtenstein)

 Subgroup 2: Monolithic zirconia (IPS e.maxZirCAD MT) (IvoclarVivadent: Schaan, Liechtenstein)

 Subgroup 3: Monolithic zirconia (IPS e.max ZirCAD Prime) Ivoclar Vivadent: Schaan, Liechtenstein)

**Figure 1:**
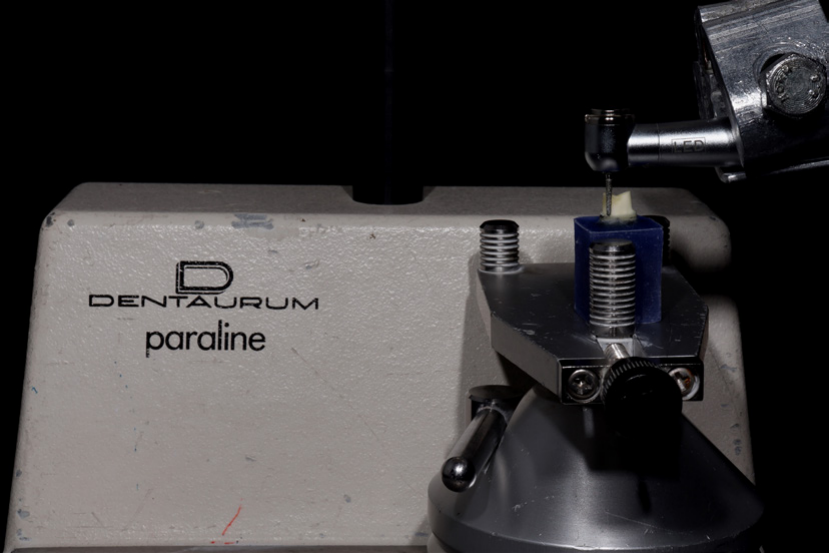
Preparation of teeth using a modified dental surveyor

**Figure 2:**
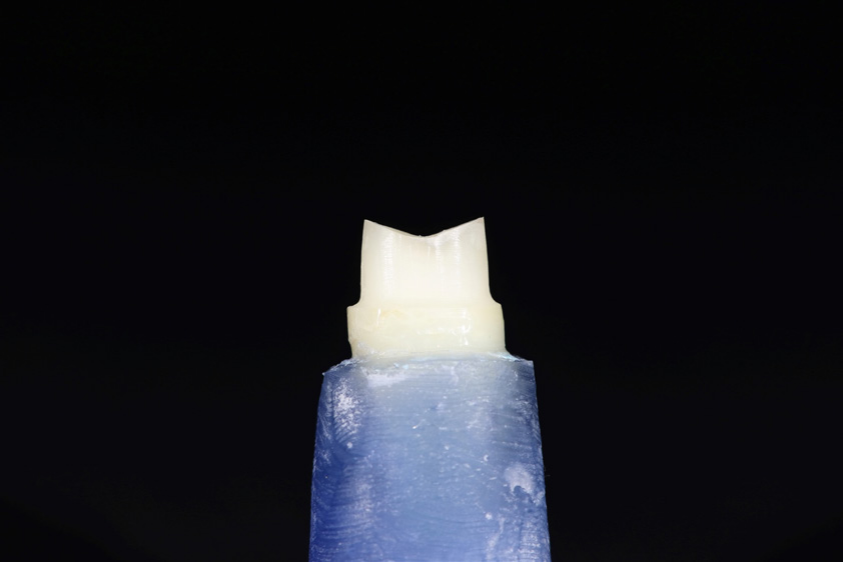
Prepared tooth sample from group A

 For standardization, a dental surveyor was used to prepare all the samples (Figure 1). The surveyor’s vertical arm was adjusted to hold a high-speed handpiece (Foshan Shengling Medical Apparatus, China) to ensure parallelism between the bur’s long axis and the tooth’s long axis, verified with a protractor.^13^ All teeth were prepared with an axial height of 4 mm measured from the mesial surface to the finish line 1 mm above the CEJ. The chamfer margin design (0.8 mm in depth) for group A teeth was prepared with a round-end tapered fissure diamond bur (6856 314 016, Komet, Germany) with a total convergence angle of 6º (Figure 2).

**Figure 3:**
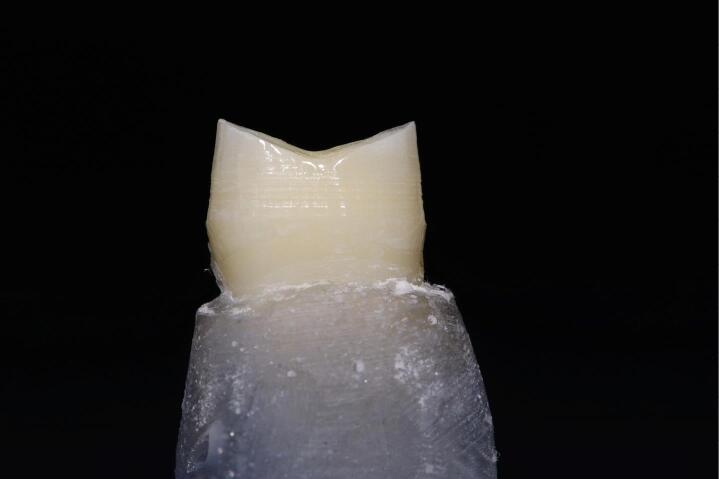
Prepared tooth sample from group B

 In group B, the vertical margin was prepared using a round-end tapered diamond bur (851-012C-FG, NTI, Germany) with a 4º total convergence angle (Figure 3). The barrel-shaped trapezoid diamond bur (811LG.314.037, VERDANT, Poland) was used for planar occlusal reduction of approximately 1.5–2 mm in both groups. A digital caliper was used to check all measurements.

 CEREC Omnicam intraoral scanner (Sirona, Germany) was used to take a digital impression for every tooth. The crowns were then machined out of zirconia blanks (IPS e.max ZirCAD LT, MT, and Prime; Ivoclar Digital, Germany) with a 5-axis milling unit (In-Laboratory MC 5 Milling Machine, Sirona, Germany) using Sirona inLab CAD 20.0 software. The milled crowns were sintered at 1500 °C in an InFire HTC Speed Sintered Furnace (Sirona, Germany) to keep their original color, strength, and size.

 Glaze paste (FLUO Ivocolor; Ivoclar Vivadent, Liechtenstein) was used to brush the crowns. The glaze firing/crystallization was performed in the Programat P500 furnace (Ivoclar, Germany) at 710 °C for 18 minutes. The interior surfaces of all crown restorations were sandblasted for 15 seconds with 1 bar at a distance of 10 mm and aluminum oxide particles measuring ≤ 50 μm to create a rough and retentive surface with a sandblasting machine (Renfert, Germany) to promote mechanical interlocking between the luting and zirconia.^14^

**Figure 4:**
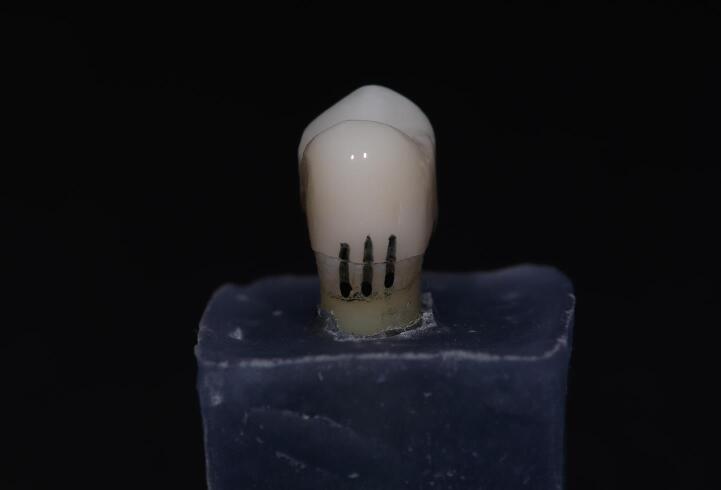
Points of measurement for the marginal gap

**Figure 5:**
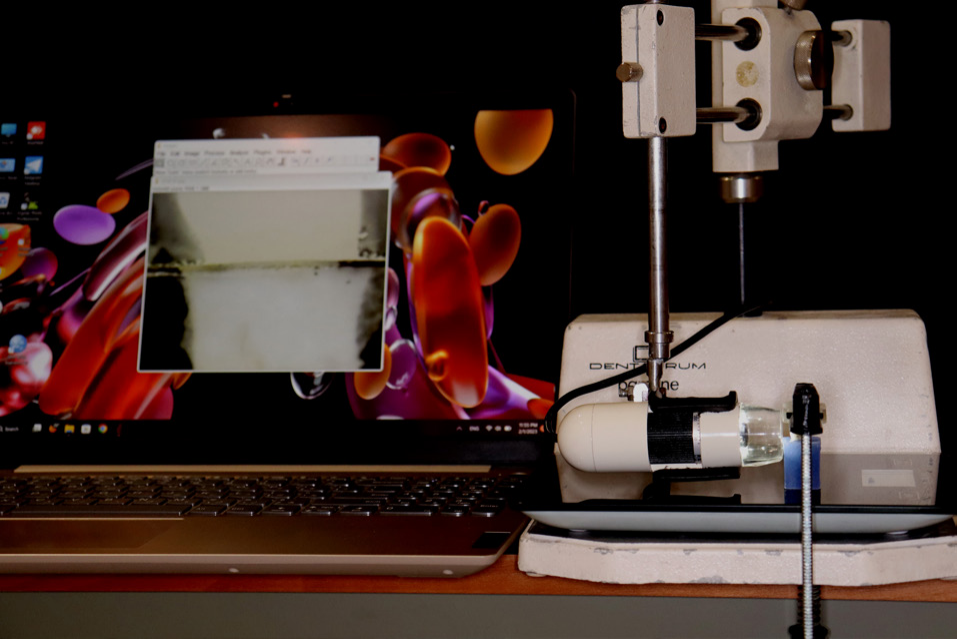
Measurement of marginal gaps by a digital microscope attached to a computer

 The vertical marginal gap for every crown was determined by employing a Dino-lite digital microscope at × 230 magnification and ImageJ software, which corresponded with the marginal gap definition byHolmes et al^15^: “perpendicular distance from the margin of the restoration to the margin of the tooth preparation.” Mid-buccal, mid-mesial, mid-distal, and mid-palatal were the four points on each surface of the specimen where the measurement was taken” (Figure 4). For each sample, sixteen measurements were taken, and the mean of all these measurements was calculated to represent the pre-cementation marginal gap (Figure 5). The same operator performed each measurement three times to reduce errors as much as possible.^16,17^

**Figure 6:**
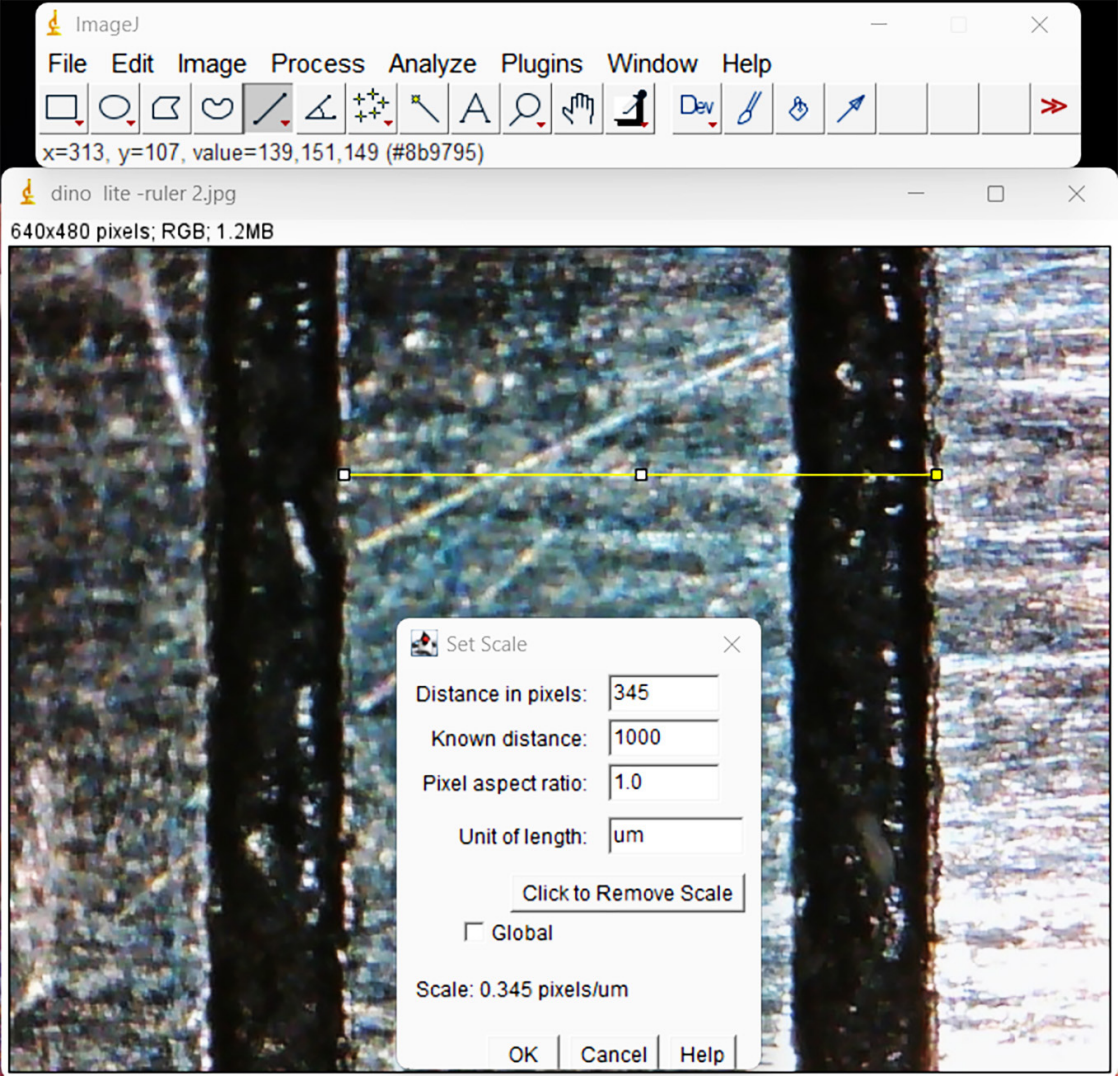
Calibration process of the ImageJ software

 The digital microscope was used to capture a snapshot of 1 mm of a ruler at a zoom of 230x to convert these measurements from “pixels” to “micrometers” for image calibration. The image was then opened in the ImageJ program, and a (straight line) tool was used to create a line corresponding to a known distance of 1 mm. The analyze option was then selected from the main menu at the same microscope calibration and magnification, and the set scale was opened to convert all calculated pixel readings to “μm.”^18^ The known distance was entered into the dialog, along with the unit of measurement (1000 and μm, respectively). The pixel section was filled in automatically with a distance defined by the length of the selected line^19^ (Figure 6).

 The marginal gap was measured after cementation following the same procedures as the pre-cementation measurement. The crowns were subsequently attached to their respective teeth with a self-adhesive resin cement (TheraCem BISCO, USA). Finger pressure was used for seating the crown before a 5-kg vertical load was applied for 6 minutes with a (custom-made cementation apparatus).^20^ All the samples were immersed and kept in distilled water at 37 °C for one day (24 h).^21^

 Statistical evaluation was performed using SPSS 27. The Shapiro-Wilk test was utilized to evaluate the variables’ normal distribution. ANOVA was utilized to determine the significance of the mean difference in fracture strength within groups. An independent t-test was used to determine the significance of the fracture strength mean difference between groups.

## Results

 The results of the Shapiro-Wilk test demonstrated that the data were distributed normally with a *P* value < 0.05. Table 1 presents descriptive data such as means and maximum and minimum vertical marginal gaps in (μm) for the two groups and their subgroups pre- and post-cementation, including standard deviation. Pre-cementation, subgroup B1 (vertical preparation design, ZirCAD LT material), had the lowest mean value of marginal gap (16.123 μm). In comparison, the highest mean value of marginal gap (25.159 μm) was recorded in subgroup A2 (chamfer preparation design, ZirCAD MT material).

**Table 1:** Descriptive statistics of the vertical marginal gap in (μm)

**Main group**	**(A) Chamfer preparation**			**(B) Vertical preparation**		
**Subgroup**	**(A1) LT**	**(A2) MT**	**(A3) Prime**	**(B1) LT**	**(B2) MT**	**(B3) Prime**
**Pre-cementation marginal gap**						
Min	20.294	21.897	20.65	14.294	15.212	14.863
Max	28.063	27.196	28.533	18.034	18.266	17.785
Mean	23.641	25.159	23.814	16.123	16.360	16.382
SD	2.851	1.801	2.491	1.572	1.166	0.973
**Post-cementation marginal gap**						
Min	34.719	34.172	33.326	24.691	25.369	22.148
Max	37.358	38.165	36.891	29.752	29.863	28.898
Mean	36.022	36.833	35.450	27.700	28.116	26.689
SD	0.775	1.309	1.319	1.671	1.471	2.230

 Meanwhile, there is a general increase in the mean values of the marginal gap among all groups post-cementation. Subgroup B3 (vertical preparation design, ZirCAD Prime material) exhibited the lowest mean value of the marginal gap (26.689 μm), while the highest mean value of the marginal gap (36.833 μm) was recorded in subgroup A2 (chamfer preparation design, MT material).

 The marginal gap was compared between the groups by performing an independent t-test at a significance of 0.05, as shown in Tables 2 and 3. The results of the current study demonstrated a statistically significant difference in both pre - and post-cementation marginal gap between both groups (*P* < 0.05).

**Table 2:** Independent t-test for comparing the marginal gaps between groups (effect of preparation design) pre-cementation

**Subgroups**		**Mean difference**	**t-value**	**Df**	***P*** ** value**
A1	B1	7.518	6.531	14	0.000
A2	B2	8.799	11.599	14	0.000
A3	B3	7.432	7.861	14	0.000

**Table 3:** Independent t-test for comparing the marginal gaps between groups (effect of the preparation design) post-cementation

**Subgroups**		**Mean difference**	**t-value**	**Df**	***P*** ** value**
A1	B1	8.321	12.779	14	0.000
A2	B2	8.716	12.519	14	0.000
A3	B3	8.761	9.566	14	0.000

 ANOVA with a significance level of 0.05 was carried out to compare the marginal gap within a group, as shown in Tables 4 and 5. Within both main groups, no significant differences were identified in both pre- and post-cementation marginal gaps.

**Table 4:** One-way ANOVA for comparing the marginal gaps within groups (effect of the material type) pre-cementation

**Groups**		**Sum of squares**	**df**	**Mean square**	**F**	***P*** ** value**
A (A1-A2-A3)	Between groups	11.050	2	5.525	0.943	0.405
	Within groups	123.048	21	5.859		
	Total	134.098	23			
B (B1-B2-B3)	Between groups	0.329	2	0.164	0.103	0.902
	Within groups	33.435	21	1.592		
	Total	33.763	23			

**Table 5:** One-way ANOVA test for comparison of the marginal gap within groups (effect of the material type) post-cementation

**Groups**		**Sum of squares**	**df**	**Mean square**	**F**	***P*** ** value**
A (A1-A2-A3)	Between groups	7.724	2	3.862	2.858	0.080
	Within groups	28.379	21	1.351		
	Total	36.103	23			
B (B1-B2-B3)	Between groups	8.620	2	4.310	1.303	0.293
	Within groups	69.490	21	3.309		
	Total	78.110	23			

 Paired t-test was used to determine any significant difference in mean values of marginal gaps post- and pre-cementation within each subgroup, as shown in Table 6. There were significant differences in each subgroup between post- and pre-cementation marginal gaps (*P* < 0.05).

**Table 6:** Paired t-test for comparing the pre- and post-cementation marginal gaps in (μm)

**Subgroups**		**Mean difference**	**t-value**	**df**	***P*** ** value**
A1	Post‒pre-cementation	12.381	10.032	7	0.000
A2	Post‒pre-cementation	11.674	18.905	7	0.000
A3	Post‒pre-cementation	11.636	16.572	7	0.000
B1	Post‒pre-cementation	11.577	21.561	7	0.000
B2	Post‒pre-cementation	11.756	16.819	7	0.000
B3	Post‒pre-cementation	10.308	12.983	7	0.000

## Discussion

 The demand for minimally invasive dentistry has led to the introduction of vertical preparation as a less intrusive approach to tooth structure restoration.^7^ To ensure standardization in production, all crowns were fabricated using the same designing software (inLab CAD 20.0), restoration parameters, and a 5-axis milling machine (inLab MC X5), which offers better marginal accuracy compared to a 4-axis milling machine.^22^

 A finishing line with a chamfer preparation design was used for horizontal preparation because many studies have shown that it produces better marginal accuracy than a shoulder finish line.^16,23^ For all prepared teeth, a planar occlusal reduction was used to minimize tooth structure removal, compromising tooth vitality.^4^ A digital microscope was used to measure the marginal gap because it is a non-destructive, direct method that does not damage the specimens. This method was also clinically applicable and the most commonly used method for measuring the vertical gap.^14,17,24^

 The most popular and clinically applicable technique for assessing the suitability of crown restorations has been considered the vertical marginal gap evaluation.^25^ The clinically acceptable vertical gap for CAD/CAM-fabricated restorations has been reported to range between 17 and 118 m.^20,26^ In this study, all vertical marginal gaps post- and pre-cementation measurements were within the clinically acceptable range.

 There was no significant difference between groups (no effect of material type) for both preparation designs, which can be attributed to the fact that all materials were from the same manufacturer and used the same parameters provided by the manufacturer, the same fabrication process (ZirCAD Labside Instructions for Use, Ivoclar, 2021), and the same CAD/CAM system and scanner. These findings are consistent with those of Att et al,^27^ who concluded that the manufacturing technique influences the marginal accuracy of CAD/CAM prostheses. According to a study,^22,28^ the CAD/CAM system affected the marginal adaptation of all-ceramic crowns fabricated using the CAD/CAM technology. Another study discovered a significant difference in accuracy between the tested intraoral scanners.^29^

 The chamfer preparation design recorded a higher pre- and post-cementation marginal gap, with a significant difference from the vertical preparation design, which recorded a lower marginal gap. This may be explained by the restoration margin being closer to the teeth when it has an acute angle at its end.^30^

 A larger gap with the chamfer finish line was most likely caused by its design, which may not have been properly scanned, resulting in less adaptation.^31^

 A chamfer finish line’s curved (concave and convex) surfaces may make it more difficult to mill the crown restoration, which would reduce the marginal fit.^32^

 The subtractive milling process is limited by the milling bur’s restricted size and shape. As a result, small details of concave shape cannot be milled with high precision.^33^

 The same findings have been reported by Comlekoglu et al^34^ and Almahdy et al,^35^ concluding that monolithic zirconia constructed with feather edge margins has better marginal accuracy than deep chamfer margin designs.

 On the other hand, Cetik et al^36^ discovered a comparable internal and marginal adaptation with the chamfer and knife-edge margins in their SEM study, which both yielded better adaptation results than the shoulder.

 Eldamaty et al^37^ discovered no statistically significant differences in the marginal accuracy of monolithic zirconia crowns with chamfer and vertical margins. However, they used metal dies instead of natural teeth as abutments used in the present study.

 Since both the cementation process and the cement play a significant role in the final discrepancy of the restoration, a marginal gap measurement of the restoration without cement cannot be considered clinically relevant.^38^ The marginal gap results after cementation showed a significantly higher mean value than before cementation, regardless of margin design. Other studies have found that cementation increases the vertical marginal gap significantly.^16,17,38^ This could be due to the hydraulic pressure created during cementation, which could push the cement upward.^38^ Another possible explanation is that the marginal spacer in the CAD/CAM system’s designing software was set to “zero,” while the radial and occlusal spacers were set to “100 µm” beginning 1 mm above the preparation margin. As a result, as the crown approaches its final position, there is no space for cement’s escape through the cervical marginal collar, resulting in a large amount of luting cement accumulating on the occlusal surface of the prepared tooth, potentially interfering with proper crown seating and increasing the marginal discrepancy.^39^ A 25-µm spacer at the marginal area of the finishing line reduces marginal and internal gaps in comparison with using zero cement space at the marginal area.^40^

 In contrast, other studies revealed that cementation did not significantly increase zirconia crowns’ vertical marginal discrepancy.^27,41,42^ The various parameters, such as the cement type, cement volume, and seating force used during cementation, may account for these conflicting findings.

 This study was carried out in vitro with natural teeth as the substrate. Natural teeth vary in size, form, and structure. Even though in vitro studies can provide controlled conditions, other aspects associated with the aging of the restoration, such as low-temperature degradation and stress-induced toughening of zirconia, which may alter the restoration behavior intraorally, were not investigated due to the in vitro experimental settings. Although measuring the marginal gap is regarded as the gold standard for assessing the adaptability of the restoration, it does not reflect the overall seating of the restoration, so measuring the internal gap is still necessary. It is also necessary to study the fracture strengths and marginal adaptations of monolithic zirconia crowns using different cement spacers, different types of cement, and different margin thicknesses.

## Conclusion

 In summary, based on the drawbacks associated with this in vitro study, four main conclusions are made.

1. The mean values of the marginal gap of zirconia crowns in all the groups were within the clinically acceptable range.
2. The mean values of the marginal gap of zirconia crowns in all the groups increased significantly after cementation.
3. The vertical preparation design resulted in a significantly lower marginal gap than the chamfer preparation design.
4. Materials’ comparisons within both preparation designs revealed comparable marginal gap results.

The above conclusions suggest using vertical preparation as a minimally invasive alternative to horizontal preparation for monolithic zirconia crowns.

## Acknowledgments

 We would like to acknowledge the support of the Department of Restorative and Aesthetic Dentistry, College of Dentistry, University of Baghdad, Iraq, for providing the necessary resources and facilities for this research.

## Competing Interests

 The authors declare no conflicts of interest related to this research.

## Ethical Approval

 The research project with project No. 503522 received ethical approval from the Research Ethics Committee of the College of Dentistry, University of Baghdad (Ref. number: 503) on March 10, 2022.

## Funding

 No external funding was received for this study.
